# Novel virulence factor Cba induces antibody-dependent enhancement (ADE) of *Streptococcus suis* Serotype 9 infection in a mouse model

**DOI:** 10.3389/fcimb.2023.1027419

**Published:** 2023-02-21

**Authors:** Pengjiang Yang, Lei Yang, Kun Cao, Qian Hu, Yuli Hu, Jun Shi, Dun Zhao, Xinglong Yu

**Affiliations:** College of Veterinary Medicine, Hunan Agricultural University, Changsha, China

**Keywords:** *Streptococcus suis*, Cba, gene knockout, ADE, immunization

## Abstract

*Streptococcus suis* (SS) is a zoonotic pathogen that affects the health of humans and the development of the pig industry. The SS Cba protein is a collagen adhesin, and a few of its homologs are related to the enhancement of bacterial adhesion. We compared the phenotypes of SS9-P10, SS9-P10 *cba* knockout strains and its complementary strains *in vitro* and *in vivo* and found that knocking out the *cba* gene did not affect the growth characteristics of the strain, but it significantly reduced the ability of SS to form biofilms, adhesion to host cells, phagocytic resistance to macrophages and attenuated virulence in a mouse infection model. These results indicated that Cba was a virulence related factor of SS9. In addition, Mice immunized with the Cba protein had higher mortality and more serious organ lesions after challenge, and the same was observed in passive immunization experiments. This phenomenon is similar to the antibody-dependent enhancement of infection by bacteria such as *Acinetobacter baumannii* and *Streptococcus pneumoniae*. To our knowledge, this is the first demonstration of antibody-dependent enhancement of SS, and these observations highlight the complexity of antibody-based therapy for SS infection.

## 1 Introduction

SS is a Gram-positive, facultatively anaerobic coccus that is a pathogen of pigs and a zoonotic agent, causing diseases such as sudden death (pigs), septic shock (humans), and meningitis (both species) (Staats et al., 1997; Wertheim et al., 2009; Gottschalk et al., 2010). Currently, 29 serotypes have been proposed for SS according to traditional serotyping methods. With the development of genotyping technology, SS has been genotyped using the capsular polysaccharide (CPS) locus, and 27 additional novel CPS synthesis (*cps*) loci (NCL) were reported(Okura et al., 2016; Zheng et al., 2017). SS1, SS2, SS7, and SS9, identified through traditional typing, are the main pathogenic serotypes in pigs (Staats et al., 1997).

Some serotypes such as serotype 1, 2, 5, 9, 14, 16, 28, and 31 can infect humans (Lun et al., 2007; Gottschalk et al., 2010; Nutravong et al., 2014; Taniyama et al., 2016), and therefore, SS has serious implications for public health safety.

More than 100 virulence factors of SS are known (Segura et al., 2017), around one-third of which are surface proteins that function outside the cytoplasm, and these surface proteins have numerous functions, including adhesion to and invasion of host cells and tissues, evasion of immune responses and biofilm formation, etc (Fittipaldi et al., 2012). Among these are the surface proteins containing the conserved LPxTG motif (LPxTG, Leu-Pro-x-Thr-Gly) (Bonifait et al., 2010). Cbp40 (collagen-binding protein 40) of SS2 and Cnm and Cbm of *Streptococcus mutans* (collagen-binding protein of *Streptococcus mutans*) enhance bacterial adhesion and colonization, which are important pathogenic factors for bacteria (Sato et al., 2004; Nomura et al., 2012; Zhang et al., 2013; Araujo Alves et al., 2018). Cba (collagen-binding adhesive, GenBank: AER20303.1), a collagen adhesive of SS, is a homolog of the afore-mentioned virulence factors and may also has the potential to function as a virulence factor.

To further understand the mechanism of SS-host interaction, an in-depth study of the virulence genes and proteins in SS is necessary. Bacterial surface proteins containing the LPxTG motif associated with bacterial virulence, such as the Cbp40 (Zhang et al., 2013), HP0197 (Zhang et al., 2009), and Sao (Li et al., 2007) proteins of SS, the SpaA protein family of *Erysipelothrix insidiosa*, and the Omp85 protein family of gram-negative bacteria (Torres et al., 2018), are highly immunogenic and potentially valuable for the development of relevant vaccines. In a preliminary study, strain DN13 of SS9 was isolated from a pig that died of septicemia in Chenzhou, Hunan Province, China. We found that its virulence was significantly enhanced when it was sub-cultured in ICR mice up to the 10th generation (SS9-P10). To explore the mechanism underlying this enhanced virulence, we comparatively analyzed the genome sequences of DN13 and SS9-P10 strains and in addition to the previously identified transcriptional regulator SrtR (Hu et al., 2018), we found that Cba containing the LPxTG motif showed multi-site mutations in SS9-P10 compared to the parental strain DN13, which could be another potential virulence-related factor for SS9-P10. In the present study, we report the impact of the *cba* gene on virulence of SS by growth assays, *in vitro* adhesion and phagocytosis assays, and finally by *in vivo* mouse infection assays. Our data suggest that Cba of SS9-P10 is a novel virulence factor. Simultaneously, we cloned the gene fragment for recombinant expression and immunized ICR mice with it to investigate its potential as an immunoprotective antigen of SS. However, mice immunized with the Cba protein were more sensitive to SS, with a higher mortality rate than that in the control group. This phenomenon was beyond our expectations.

## 2 Materials and methods

### 2.1 Bacterial strains, plasmids, and cultures

The strains and plasmids used in the present study are listed in **Table 1**. The background and culture conditions for DN13 and SS9-P10 were based on our previous report (Hu et al., 2018). Briefly, SS*-*related strains were cultured in brain-heart infusion (BHI) medium; DH5α and *Escherichia coli*-related strains were cultured on Luria-Bertani (LB) medium at 37°C and shaking at 180 rpm. When necessary, antibiotics were added for SS transformations at the following concentrations: 50 μg/mL for spectinomycin, 8μg/mL for chloromycetin. Antibiotic concentrations for E. coli were 25 μg/mL of chloromycetin, 50 μg/mL of spectinomycin and 50 µg/ml of kanamycin.

**Table 1:** Strains and plasmids used in this study.

Name	Characteristic	Source or reference
SS9-P10	DN13 was transmitted to the 10th generation in mice	Hu et al., 2018
BL21 (DE3)	*Escherichia coli* engineer bacteria for the expression of recombinant protein	Tsingke Biotechnology Co., Ltd
DH5α	*E. coli* engineer bacteria for the expression of recombinant protein	Tsingke Biotechnology Co., Ltd
pSET6s	Thermosensitive suicide vector for the construction of an isogenic mutant in SS; *spc^R^*	Takamatsu et al., 2001
pSET1	*E. coli-*SS shuttle cloning vector; *cm^R^*	Takamatsu et al., 2001
pET28a (+)	Plasmid expressing recombinant protein	Tsingke Biotechnology Co., Ltd

cm^R^, chloramphenicol resistance; spc ^R^, spectinomycin resistance.

### 2.2 Construction of the SS9-P10 *cba* gene knockout strain and its complementary strain

The construction of non-polar knockout mutants and complementation strains was performed as previously reported (Hu et al., 2018). Briefly, SS9-P10 genomic DNA was used as a template to amplify the upstream and downstream homologous arms of the *cba* gene using primers Cba-LF/Cba-LR (enzyme cut site: HindIII, SalI) and Cba-RF/Cba-RR (enzyme cut site: BamHI, EcoRI) to obtain *cbaL* and *cbaR* fragments, respectively. The pSET4_S_ plasmid was used as a template to amplify the *spc* gene fragment using primers Spc-F/Spc-R (enzyme cut site: SalI, BamHI). The *spc*, *cbaR*, and *cbaL* fragments obtained by PCR were sequentially cloned into the pSET6s vector and verified using Sanger sequencing at BIOSUNE Biotechnology Co., Ltd (China) to obtain a pSET6s-Δ*cba* knockout plasmid. Competent streptococcal cells were prepared under electroporation conditions according to a previous report (Takamatsu et al., 2001). After introducing pSET6s-Δ*cba* into SS9-P10, double-crossover *cba* deletion mutants (SS9-P10:Δ*cba*) were screened. The complementary DNA fragments of the *cba* by PCR using primers CbaF and CbaR. The resultant product was then inserted into pSET1 at BamHI/PstI sites to generate pSET1-*cba*. This plasmid was used to introduce a functional *cba* gene into SS9-P10:Δ*cba* to generate complementing strains SS9-P10:CΔ*cba*. Empty pSET1 was introduced into SS9-P10 as a control to exclude the effect of the vector. Primer sequences are listed in **Table 2**.

**Table 2:** Related primers of *cba* mutation strain.

Primer Name	Primer sequences (5′ - 3′)	Function
Cba-LF	CCCCAAGCTTAGTGGTACTGGAAACCGAATT	Amplification of *cba* upstream homologous arm
Cba-LR	CGCGTCGACCAGTCTAATTGTCTTCTTAAAAC	
Cba-RF	GCGGGATCCTACCGAGGAGCCAATGACTA	Amplification of *cba* downstream homologous arm
Cba-RR	CGGAATTCCGGTTATACTCAGGTAATGCT	
Spc-F	CGCGTCGACGTGAGGAGGATATATTTGAATAC	Amplification of *spc* resistance gene fragment
Spc-R	GCGGGATCCTTATAATTTTTTTAATCTGTTATTTAAA	
Cm-f-S	CGCGTCGACATGAACTTTAATAAAATTGATTTAGA	Plasmid-specific primers for pSET6s and pSET1
Cm-r-B	GCGGGATCCTTATAAAAGCCAGTCATTAGGC	
ORF Cba-F	ATGATTGGTGGAGCATACGTTG	*cba* gene detection primer
ORF Cba-R	TTAAGCATTCTTAGATTTACGG	
pSETa	GGTGAAAACCTCTGACACATGC	pSET1 plasmid detection primers
pSETb	CGTTACCCAACTTAATCGCCTT	
Cba-F	CGCGAATTCATGTTTGAAATTCAAGGTCGCGGATA	Cloning upstream primers of *cba* fragment
Cba-R	CGCGTCGACTTAGAGCCGTTTTTCTAGTTTTC	Cloning Downstream Primers of *cba* fragment
CbaF	aaaa CTGCAGttaaatatcaatctataattaaag	For amplification of complementary DNA fragments of *cba*
CbaR	cg GGATCCttaagcattcttagatttacggt	

### 2.3 Bacterial growth assay

SS was resuscitated and incubated at 37°C for more than 8 h (or overnight). The bacterial culture was subsequently diluted with fresh BHI medium to an OD600 of 0.1 and incubated at 37°C for 12 h. Growth was observed every hour to determine growth characteristics.

### 2.4 Characterization of *cba* on bacterial virulence *in vitro*

#### 2.4.1 Biofilm formation assays

The capacity for biofilm formation was tested using the protocol as reported previously (Meng et al., 2011). The overnight cultures were diluted and 200μL of each strain diluent (at OD600 of 0.1) was added to 24-well microplates (each well containing 1.8ml BHI) and incubated for 72h at 37°C. The wells were then washed three times to remove free-floating planktonic bacteria. After drying in air, the biofilms were stained with 500μL of 0.1% crystal violet for 30 min. After washing with ddH_2_O, the wells were dried for 2h at 70°C. After adding 500μL of 33% glacial acetic acid to each well, the microplates was placed on a shaker for 30min to elute the biofilm-bound dye, and the biofilms were quantified by recording the absorbance at 595 nm.

#### 2.4.2 Bacterial adhesion assays

Adhesion assay of bacteria to the porcine kidney 15 (PK15) cells were performed as described previously with some modifications (Zhang et al., 2015). Briefly, PK15 cells were seeded into the wells of 24-well microplates and cultivated in RPMI 1640 (containing 10% FBS). The bacteria (SS9-P10, SS9-P10:Δ*cba* and SS9-P10:CΔ*cba*) were added to PK15 monolayers at a multiplicity of infection (MOI) of about 100:1, with triplicate wells for each strain. After 2h incubation at 37°C and 5% CO_2_, the wells were washed four times with phosphate-buffered saline (PBS) and then lysed with ice-cold ddH_2_O on ice for 20 min. The number of cell-adherent bacteria was determined by plating dilutions of the lysate on BHI plates. Adhesion is defined as CFU adhered to the cells/CFU in original inoculum × 100%. The experimental procedures were repeated three times independently.

## 2.4.3 Anti-phagocytosis assays

To evaluate the anti-phagocytic ability of the three strains. The assay was performed as the previous description with some minor modifications (Wang et al., 2019). RAW264.7 cells were cultured at 37°C and 5% CO_2_ overnight in DMEM with 10% FBS to form monolayers in 24-well plates. Then, the culture medium was discarded, wells were washed with PBS, and the bacteria were added to the wells at MOI of 10:1. The plates incubated for 1h in a CO_2_ incubator at 37°C. The wells were then washed four times with sterile PBS to remove non-adherent bacteria. DMEM containing ampicillin (100μg/ml) was then added to the wells and incubated for 1h at 37°C and 5% CO_2_. Subsequently, the macrophage was lysed with ice water after thrice times washing with PBS. Serial twofold dilutions of the cell lysate was plated on BHI agar plates. The assay was performed three independent times.

### 2.5 Characterization of *cba* on bacterial virulence *in vivo*

#### 2.5.1 Pathogenicity assays in mice

SS9-P10, SS9-P10:Δ*cba*, and SS9-P10:CΔ*cba* strains were overnight cultured in fresh BHI and subcultures of these strains were grown for 4 h to mid exponential phase. After centrifuging and washing with sterile PBS twice, suspensions were 10-fold serially diluted to approximately 10^7^ CFU/ml. Twenty-four Specific Pathogen Free (SPF) ICR mice (female, 4 weeks old, were purchased from Hunan Sleek Jingda Laboratory Animal Co.) were randomly divided into 3 groups (8 mice/group) and challenged by intraperitoneal injection with the doses of 10^6^ CFU/mouse of SS9-P10, SS9-P10:Δ*cba* and SS9-P10:CΔ*cba* respectively (100 μl/mouse). Mice were monitored once a day for 14 days to determine the mortality.

#### 2.5.2 Competitive infection experiment

A competitive infection assay was performed by referring to the method reported by Jelsbak (Jelsbak et al., 2016). Briefly, four-weeks-old female ICR mice (n=8) were infected with the same concentration (1 × 10^7^ CFU) SS9-P10 and SS9-P10:Δ*cba* mixed in equal proportions, and blood was taken from the eye sockets at 24, 48, and 72 h. The collected blood was diluted with saline, spread on BHI (for culture of SS9-P10 and SS9-P10:Δ*cba* strains) and BHI *Spc^R^* plates (for selective culture of SS9-P10:Δ*cba* strains), and incubated for 18 h at 37°C in a 5% CO_2_ incubator to calculate the bacterial load, which was then calculated using the following formula to calculate the competitive infection index. The competitive index (CI) was calculated as the SS9-P10:Δ*cba*/SS9-P10 ratio of the blood count versus the SS9-P10:Δ*cba*/SS9-P10 ratio of the inoculum.

### 2.6 Preparation of Cba recombinant protein subunit vaccine

The recombinant *cba* was expressed by an *E. coli* expression system with a pET-28a (+) vector as described in “Molecular Cloning: A Laboratory Manual.” Briefly, the *cba* gene fragment was amplified from the genomic DNA of SS9-P10 by PCR and cloned into pET-28a (+). *E. coli* strain BL21 (DE3) was used to express the recombinant Cba protein induced by isopropyl β-D-1-galactoside (IPTG). The recombinant protein was purified using HisTrap HP columns (GE Healthcare Life Sciences, USA). The subunit vaccine was formulated by mixing 200 µg/mL of recombinant Cba (rCba) 1:1 with emulsified Montanide ISA 201 adjuvant (Seppic, France).

### 2.7 Mice active immunization and chellenge assays

Sixteen ICR mice (4 weeks old) were divided into two groups (immunized group and control group) of eight mice each. Each mouse in the immunized group was intramuscularly injected with 100 μL of subunit vaccine and the control group was immunized with an equal volume of Montanide ISA 201 adjuvant. The immunization was performed with two injections with a two-week interval. Mice in the immunized and control groups were infected with SS9-P10 at a dose of approximately 1 × 10^7^ CFU/mouse 7 days after the second vaccination, and the mortality rate was counted. To ensure the reliability of the results, two challenge protection experiments were performed.

### 2.8 Determination of antibody serum levels

Serum was from mice collected on days 14, 21, 28, and 35 days after the first immunization, respectively. Three mice from each group were sampled at random on each date. Serum antibody titers were determined by indirect ELISA (Yi et al., 2021). Briefly, the 96-well microplates were coated with purified recombinant rCba (100 ng/100 µL) overnight at 4 °C. The protein solution was then removed and 100 μL of blocking solution was added to each well, blocked at 37°C for 1 h, and washed 4 times with phosphate-buffered saline containing 0.1% Tween 20 (PBST). The serum was diluted 100 times with PBST, added to the wells, and incubated at 37°C for 30 min. Following additional washings, goat anti-mouse IgG-HRP (Sera Care, USA) was added to the wells at 1:12,800 dilution. The absorbance at 450 nm was measured using a microplate reader.

### 2.9 Mice passive immunization and chellenge assays

Serum samples were collected from mice on days 7 day after the second immunization and were stored at 4°C. Sixteen ICR mice (6-week-old) were randomly divided into 2 groups (n=8 in each group). For the first group, each mouse was immunized by an intraperitoneal injection 100 μL antiserum. The second group served as a control group and mice received an intraperitoneal injection 100 μL of PBS. Twenty-four hours later, mice of each group were challenged with a dose of 1×10^7^CFU of SS9-P10/mouse by intraperitoneal injection. Mice were monitored once a day for 14 days to determine the mortality.

### 2.10 Bacterial load in the blood and tissues

To assess whether rCba immunization affects the distribution of SS in blood and organs, we injected 1 × 10^7^ CFU of SS9-P10 intraperitoneally into groups of mice (n = 10)(The immunization experimental details were the same as those described in the”Active immunization “ section). And all the challenged mice were euthanized 24h later and peripheral blood was collected from the mice, then the heart, liver, lungs, spleen, and kidneys of each mouse were collected under aseptic conditions. The serial tenfold dilutions of the tissue homogenate and blood were transferred to agar plates, the plates were incubated at 37°C for 24 hours and the number of CFUs was determined.

### 2.11 Histopathological analysis

The livers, lungs, kidneys, and spleens of dead mice in immunized and control groups were sampled. The tissues were preserved in 4% paraformaldehyde solution, and histological sections were prepared using routine methods and stained with hematoxylin and eosin.

### 2.12 Statistical analysis

Graphpad Prism version 6.0 for Windows (Graphpad Software, USA) was used to analyze the growth of bacteria and survival of mice. Unless otherwise stated, the data are expressed as mean ± standard deviation. The survival rates among the related groups were compared by logarithmic rank (Mantel-Cox) test. ^*^ *P* < 0.05, ^**^ *P* < 0.01 and ^***^ *P* < 0.001 were considered significant, extremely significant and most significant, respectively.

## 3 Results

### 3.1 Growth characteristics of mutant strains

**Figure 1:**
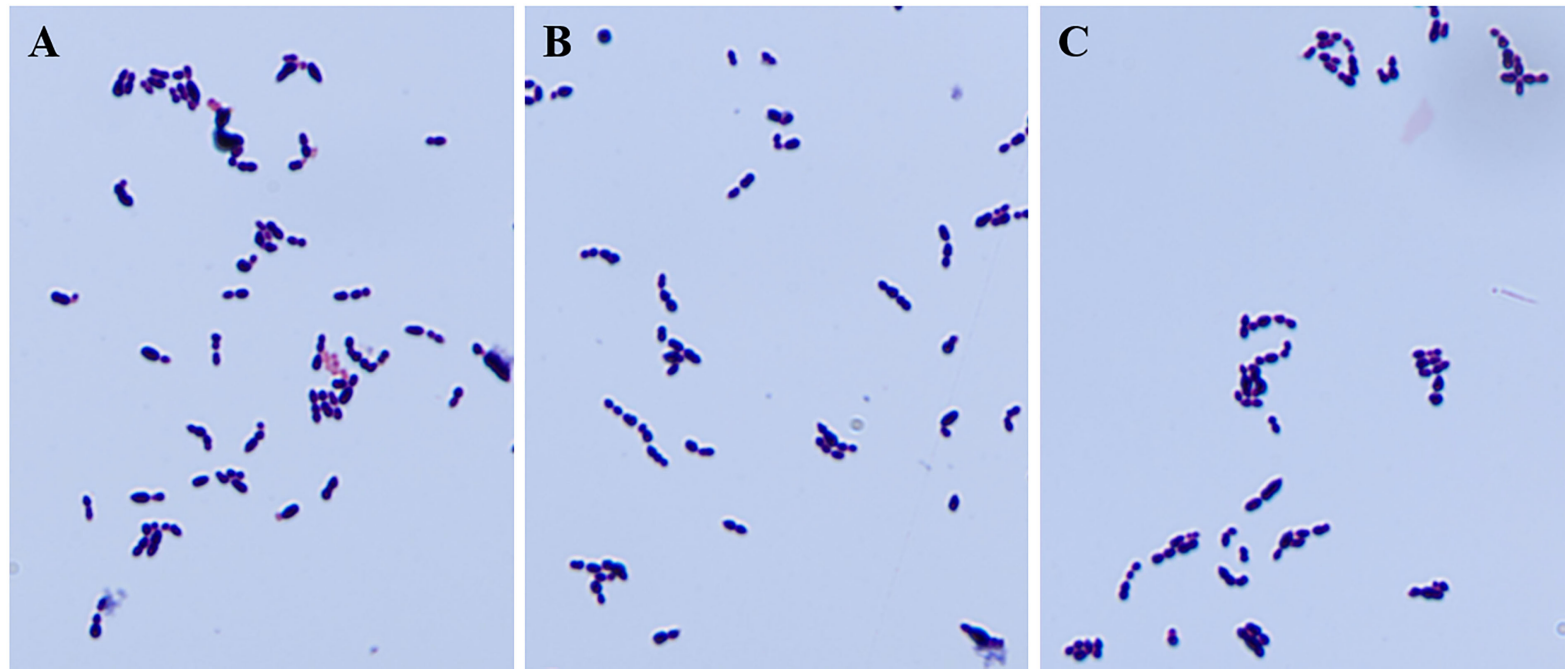
Gram staining of strains before and after deletion of *cba* gene and complementary deletion. The knockout of the *cba* gene does not affect the gram staining properties and appearance of SS. **(A-C)** are the results of microscopic examination of the gram staining of SS9-P10, SS9-P10:Δ*cba*, and SS9-P10:CΔ*cba*, respectively.

**Figure 2:**
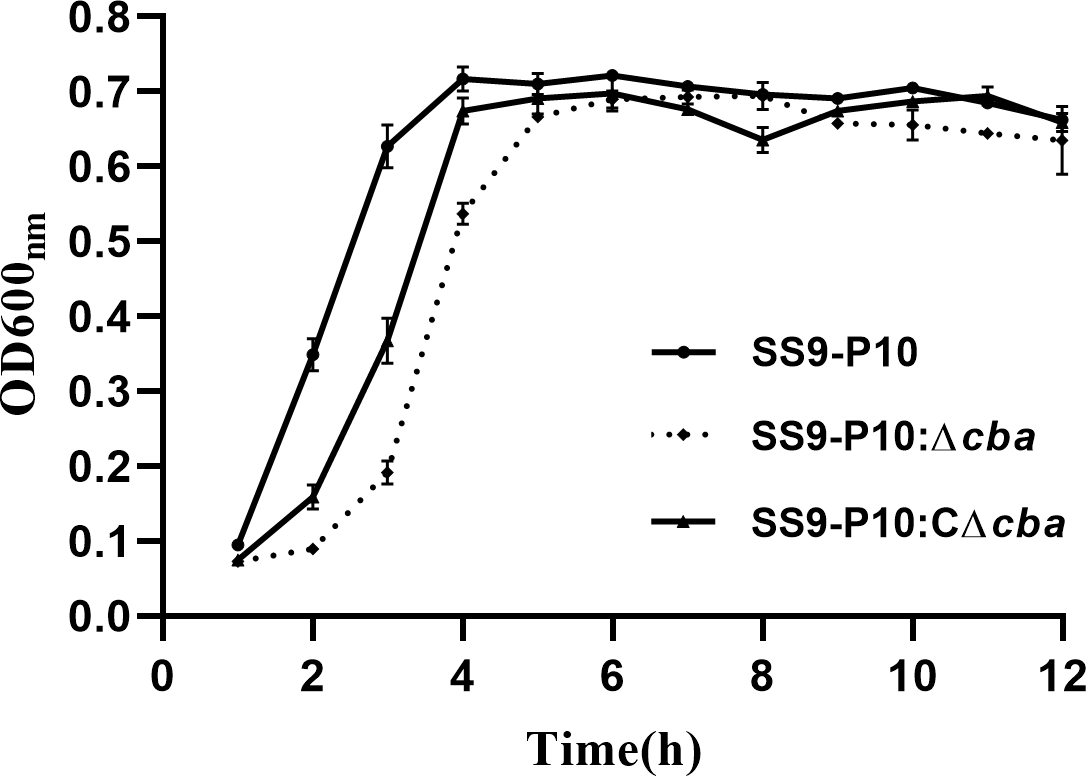
Growth curve before and after *cba* gene deletion, and the survival curve of mice during a challenge. Deletion and deletion complementation of the *cba* gene does not affect the growth of SS. All three strains entered the logarithmic growth phase after 2 h and reached the plateau phase after about 4 h.

To identify the possible role of *cba* in SS9-P10, we constructed a *cba* mutant strain (SS9-P10:Δ*cba*) and its complementary strain (SS9-P10:CΔ*cba*). The effect of *cba* deletion on general biological characteristics was evaluated. The results show that, no significant differences were observed in the morphology (**Figure 1**) and growth characteristics (**Figure 2**) of SS9-P10, SS9-P10:Δ*cba*, and SS9-P10:CΔ*cba*.

### 3.2 Characterization of *cba* on bacterial virulence *in vitro*

**Figure 3:**
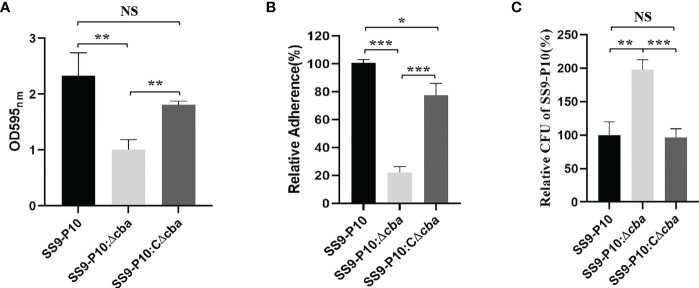
Virulence assessment *in vitro*. **(A)** Biofilm biomass was expressed as crystal violet optical density (OD540nm). **(B)** The adherence rate of SS9-P10 is significantly higher than SS9-P10Δ*cba*. **(C)** A total of 5.0×10^5^ Raw264.7 cells were infected separately with the SS9-P10:Δ*cba*, SS9-P10:CΔ*cba* or SS9-P10 at an MOI of 10:1 for 1 **(h)** CFUs of phagocytosed bacteria recovered from macrophages were examined. SS9-P10:Δ*cba* were more susceptible to phagocytosis, whereas SS9-P10:CΔ*cba* recovered the phenotype to the level of SS9-P10. Data are expressed as the mean ± SEM of three independent experiments performed in triplicate (*P<0.05; **P<0.01; ***P<0.001; ^NS^p>0.05 ***P<0.001).

The deletion of the *cba* gene significantly reduced the ability of SS to form biofilms (**Figure 3A**), adhesion to host cells (**Figure 3B**) and phagocytic resistance to macrophages (**Figure 3C**) compared with the SS9-P10 and SS9-P10:CΔ*cba* strains.

### 3.3 SS9-P10*cba* gene knockout strains attenuate pathogenic effects in mice

**Figure 4:**
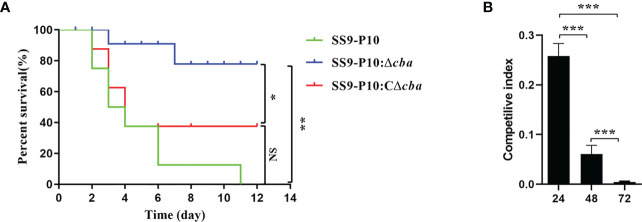
Virulence assessment *in vivo*. **(A)** Comparison of survival rates using the Log-rank (Mantel-Cox) test.Using a mouse model of infection, approximately 10^6^ colony forming units (CFUs) of SS9-P10, SS9-P10:Δ*cba* or SS9-P10:CΔ*cba* in 100 µL of sterile phosphate-buffered saline (PBS) was used to challenge a group of mice *via* an intraperitoneal injection. Survival was observed daily for 12 days. The *cba*-expressing strains were more virulent than their *cba*-deficient counterparts (SS9-P10 vs. SS9-10Δ*cba* and SS9-P10:CΔ*cba*, n=8, ^*^P < 0.05; ^**^P <0.01; ^NS^p>0.05). **(B)** Competilive index analysis between the SS9-P10:Δ*cba* and SS9-P10 in ICR mice. Data were analyzed with the Kruska-Wallis multiple comparison test (n = 8) and presented with mean ± SD (^***^P<0.001).

The pathogenicity of SS9-P10, SS9-P10:Δ*cba and* SS9-P10:CΔ*cba* in mice varied considerably. Mice in SS9-P10 challenge group was the first to show clinical signs of the disease, followed by SS9-P10:CΔ*cba* group and finally the SS9-P10:Δ*cba* groups, and all of the mice infected with SS9-P10 or SS9-P10:CΔ*cba* displayed severe symptoms, such as unkempt coat, slow response, and purulent discharge from the corners of the eyes. In terms of mortality, the SS9-P10 group had a 100% mortality rate, with mice dying on the second day after the challenge and all mice dying on the eleventh day after the challenge (n = 8). SS9-P10:CΔ*cba* group had the second-highest mortality rate, with the first death also occurring on the second day after the challenge, and the mortality rate was 62.5% on the fourth day, with the surviving mice, gradually returning to normal after the fifth day (n = 8). SS9-P10:Δ*cba* group had the lowest mouse mortality rate, with the first death occurring on the third day after the challenge and only 25% mortality rate by Day 7 (n = 8); the surviving mice gradually returned to normal (**Figure 4A**). The results showed that deletion of the *cba* gene attenuates the pathogenicity of the SS9-P10 strain in mice. In addition, the competitive infection test results showed that the CI of SS9-P10:Δ*cba*/SS9-P10 was 0.308, 0.061, and 0.004 at 24, 48, and 72 h respectively and that the CI gradually decreased over time, indicating that the survival of SS9-P10:Δ*cba* in mice was significantly inhibited by SS9-P10 (**Figure 4B**). All the above experimental results demonstrating that *cba* is a novel SS9-P10 virulence factor.

### 3.4 rCba subunit vaccine stimulates strong antibody responses

**Figure 5:**
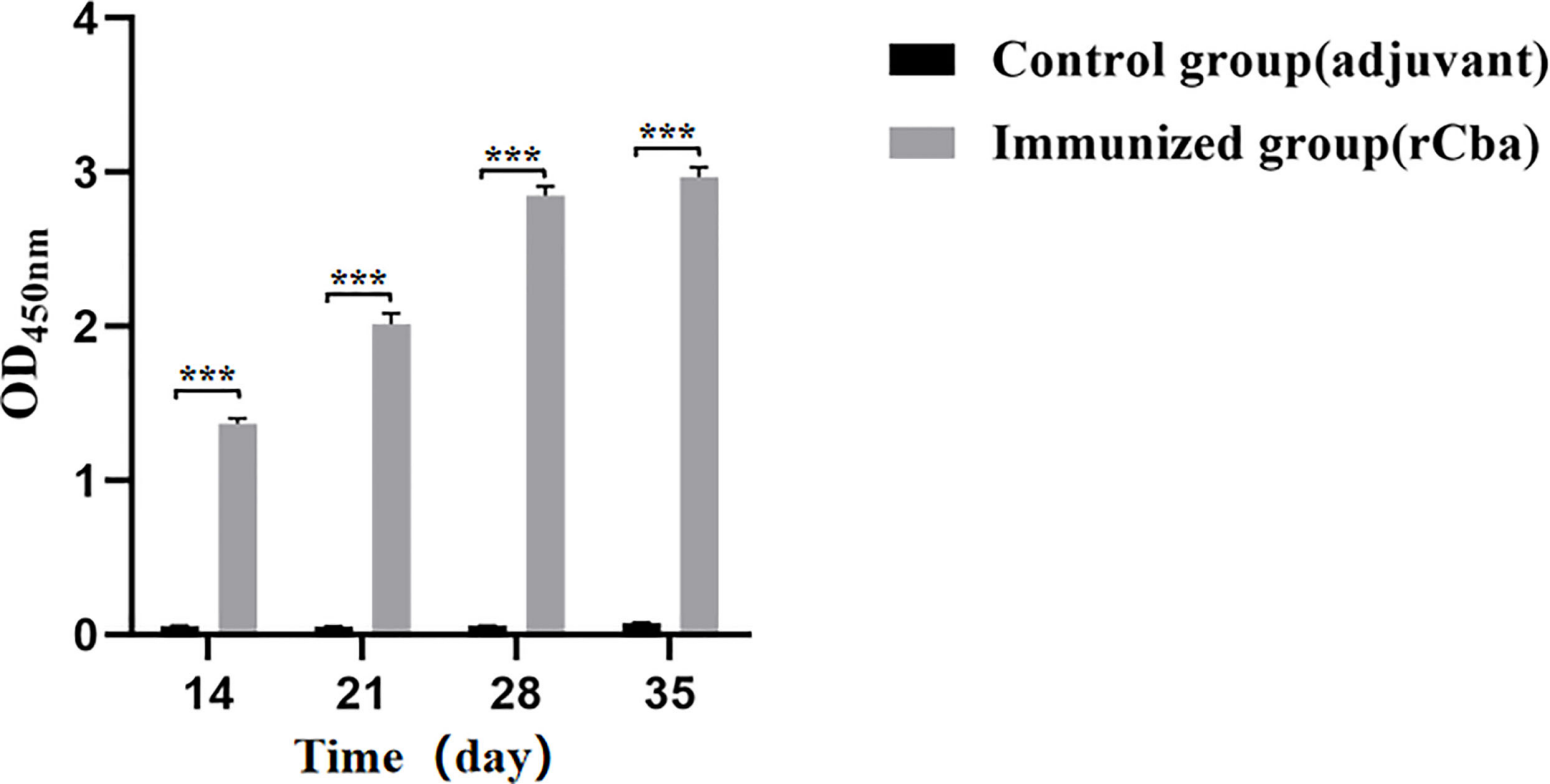
Strong immune response induced by rCba in ICR mice. Serum collected on days 7 d, 14 d, 21 d and 28 d after the first immunization. The antiserum was diluted 100 times with PBST, the goat anti-mouse IgG-HRP was diluted 1:12800, and the absorbance at OD450 nm was measured by microplate reader. (^***^P < 0.001).

IgG antibody levels were determined in the rCba immunized and control group mice using indirect ELISA. After the first immunization, there was a significant difference was noted between the immunization and control groups (**Figure 5**). The antibody level of the immunization group increased with the increase in immunization days; especially, after the second immunization, the antibody levels increased rapidly. These finding indicate the highly immunogenic nature of Cba protein.

### 3.5 Immunization of Cba recombinant protein aggravated the pathogenic effect of SS in mice

**Figure 6:**
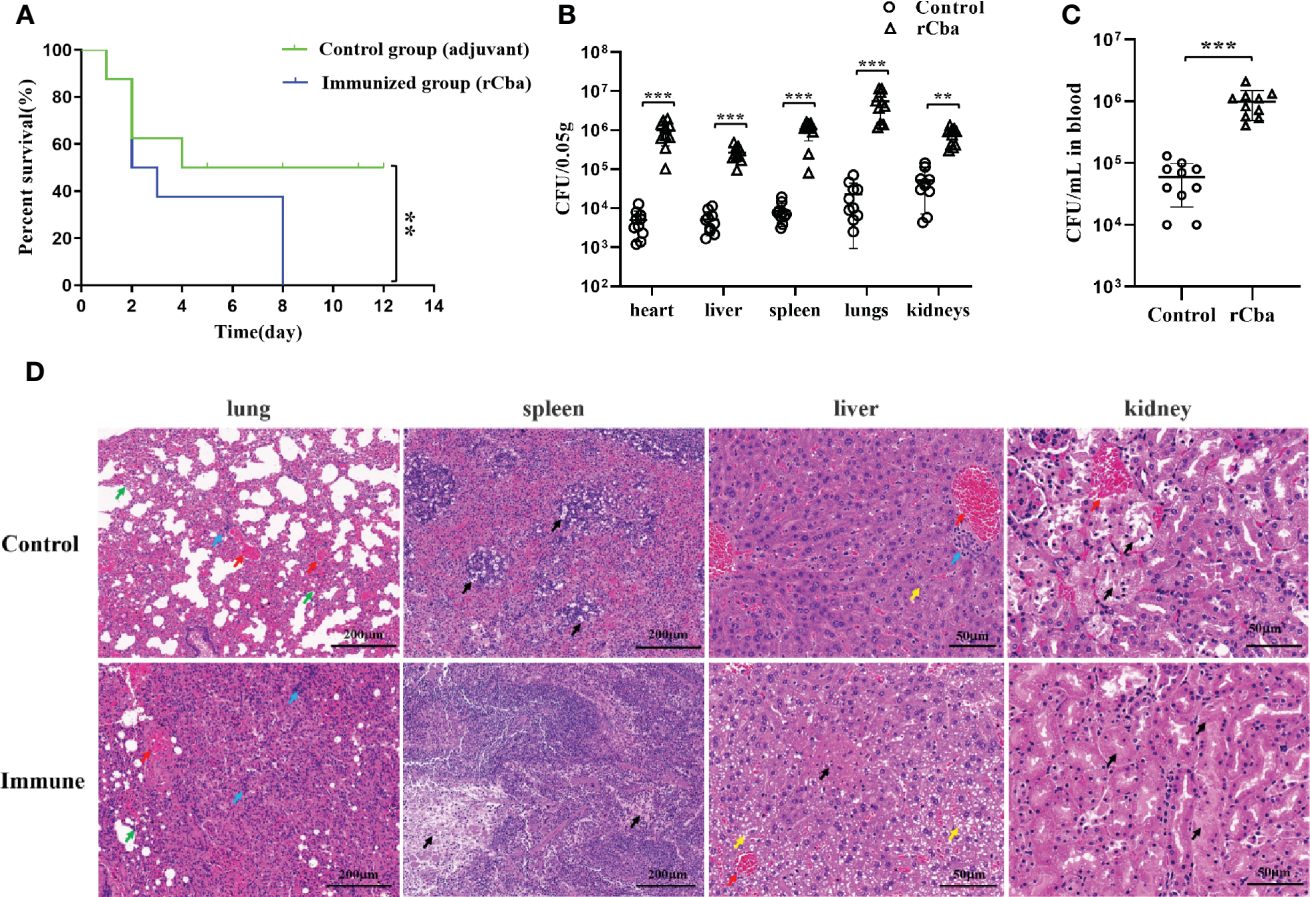
Intramuscular immunization with rCba aggravated the pathogenic effect of SS in Mice. **(A)** Kaplan-Meier survival curves of immunized mice with SS9-P10 infection. Were monitored daily for 2 weeks. The statistical significance was analyzed by log-rank (Mantel-Cox) test (n=8, ^**^P <0.01); bacterial loads in **(B)** blood or **(C)** tissue homogenate of the heart, liver, kidney, lungs, and spleen at 24 h were determined *via* colony plate counting. (n=10, ^**^ P <0.01, ^***^P<0.001); **(D)** Histopathological damage assessment of the lung, liver, spleen and kidney tissue of mice immunized with rCba and adjuvant after challenge with SS9-P10. Fatty degeneration in the liver is indicated by a yellow arrow, and hepatocytes show obvious microvesicular steatosis with a tendency to fuse similar to that in macrovesicular steatosis; hyperemia in the lung and liver is indicated by a red arrow, infiltration of inflammatory cells in the lung and liver is indicated by a blue arrow; thickening of the lung interstitium is indicated by a green arrow; and necrosis in the liver, spleen, and kidney is indicated by a black arrow.

After two immunizations, all two groups showed significant clinical signs 48 h after challenge with the SS9-P10 strain. During the next 14 days of observation, the mortality rate of the mice in immunized group increased rapidly, and no mice were left alive in the immunized group on the 8th day, while the final survival rate of the control group was 50% (4/8) (**Figure 6A**). Similar results were obtained in the repeated experiment. Further results of bacterial load assays also showed that rCba-immunized mice had higher bacterial loads in organs (**Figure 6B**) and blood (**Figure 6C**) at 24 h after infection with SS9-P10 compared to the non-immunized group.

Histopathological results revealed that the control mice showed lesions in the following major organs after being challenged with SS9-P10: The lung showed thickening of the pulmonary septum, capillary congestion in the alveolar wall, and inflammatory cells and damaged and detached epithelial cells in the alveolar lumen and bronchial lumen; the liver showed marked steatosis and fused fat droplets, a large number of inflammatory cell infiltration in the hepatic hilar area, irregular arrangement of hepatic cord, swelling and necrosis of hepatocytes, and disappearance of some hepatic sinusoids; the kidneys showed major renal lesions with renal tubular necrosis, detachment of tubular epithelial cells, and eosinophilic protein-like material in the renal tubules; and SS infection results in splenic edema with evident lymphocyte and reticulocyte damage, and reduced volume of white pulp in the infected spleen. The above pathological changes were more severe in the immunized group (**Figure 6D**).

Notably, the surviving mice of control group mice showed neurological symptoms, while mice in immunized group showed neurological symptoms at the early stage (Day 3) after the SS9-P10 strain challenge. This suggests that bacteria are more likely to cross the blood-brain barrier of immunized mice and cause neurological symptoms. Surprisingly, instead of protecting the mice, the immunization aggravated the pathogenic effect of SS.

### 3.6 Specific antiserum of rCba increases mortality in mice after challenge

**Figure 7:**
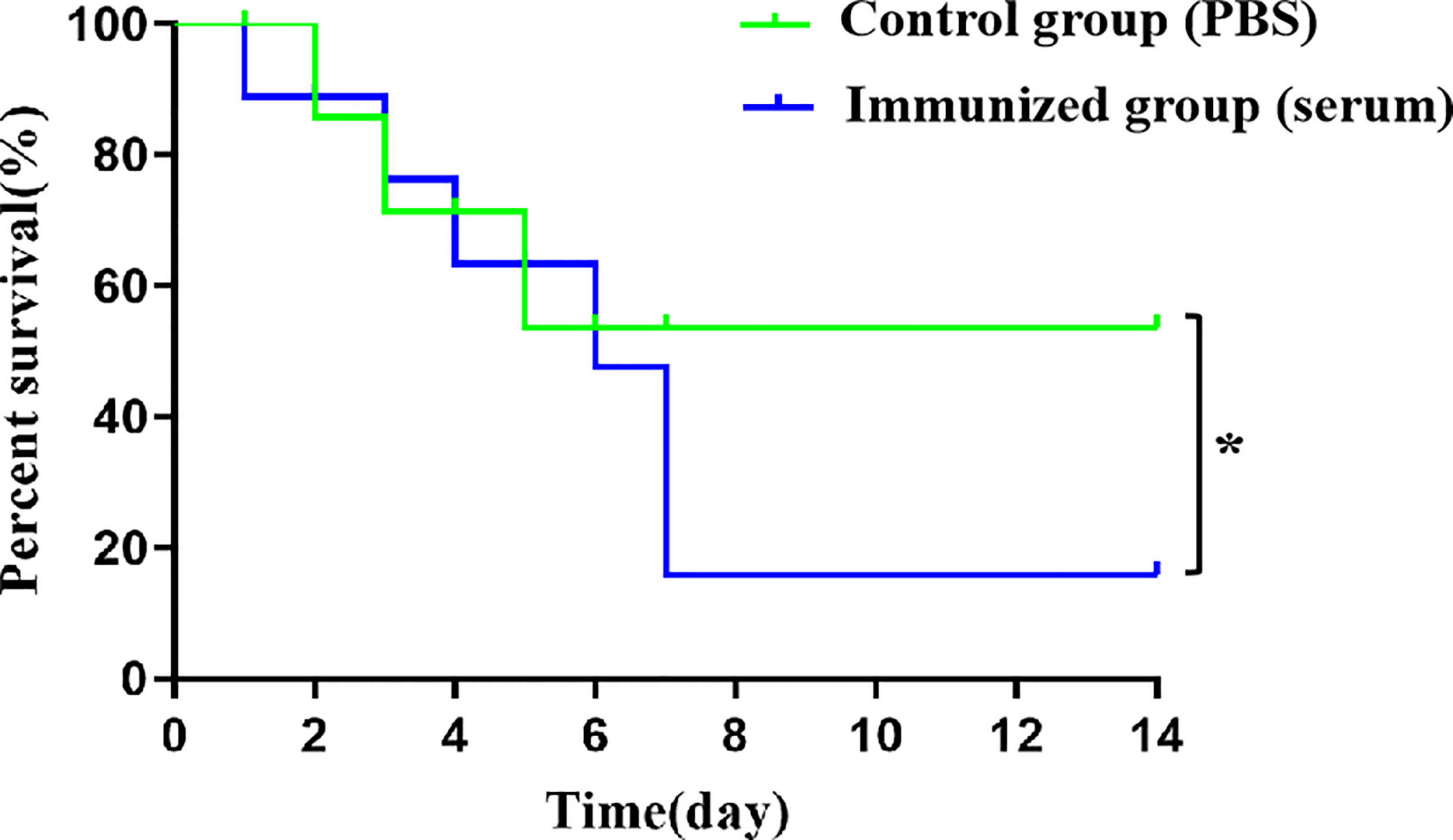
Intraperitoneal injection of antiserum improves death rate in challenge SS9-P10. Kaplan-Meier survival curves of immunized mice with SS9-P10 infection. Were monitored daily for 2 weeks. The statistical significance was analyzed by log-rank (Mantel-Cox) test (n=8, ^*^P <0.05).

In the antiserum-treated group, the first mouse died at 1 day-post-infection with SS9-P10, and the mortality rate gradually increased over time, with only one mouse surviving at 7 day-post-infection, and a final survival rate of only 12.5%.The first dead mouse in the PBS-treated group had a significantly delayed time compared to the antiserum-treated group (at 48h after infection), and 4 out of 8 (50%) mice died within 5 day-post-infection (**Figure 7**). The data suggest that serum from mice receiving active immunization likewise failed to provide protection against SS9-P10 infection and increase mortality in mice challenged with SS9-P10.

## 4 Discussion

Many cell wall-anchoring proteins containing the LPxTG motif are associated with bacterial pathogenicity in SS (Fittipaldi et al., 2012), such as SntA (Cabezas et al., 2022) Sao (Li et al., 2006), adhesins (Li et al., 2015) ApuA (Ferrando et al., 2010) and SsnA (Meurer et al., 2020). Therefore, we hypothesized that Cba containing the LPxTG motif may also be involved in SS infection of the host. Comparison of the *cba* gene sequences with the 123 SS whole genome sequences published in the NCBI database showed that 28 SS strains contained *cba* gene. The *cba* does not appear to be ubiquitous among SS strains, but further analysis of the background information of the 28 SS strains revealed that 19 of them were isolated from clinical cases in pigs or humans, and most of them (16 strains) showed varying degrees of pathogenicity to animals and humans. Detailed information on these strains see **Table S1** in the supplemental material. In parallel, we analyzed 56 SS strains(serotypes 2, 7, and 9)isolated from clinical cases preserved in our laboratory by PCR, and their results (33/56) also confirmed the prevalence of the cba gene in SS pathogenic strains.

In this study, although the presence or absence of *cba* did not affect the growth characteristics of SS9-P10, knockout of *cba* gene significantly reduced biofilm formation in strain, adhesion to host cells, phagocytic resistance to macrophages, and pathogenicity of the strain in mice, and all these virulence characterizations of bacteria both *in vivo* and *in vitro* was restored in the gene-deleted complementary strain. In addition, we compared the virulence of SS9-P10:Δ*cba* to that of the SS9-P10 in a competitive infection assay in mice. The results showed that much higher numbers of SS9-P10 were reisolated from the peripheral blood of mice compared to the numbers of SS9-P10:Δ*cba*, indicating that the mutant strain was attenuated *in vivo*. Based on these results, we hypothesize that Cba is a novel virulence factor in SS.

More than 40 protein candidates have been shown to induce either protective antibodies (through *in vitro* opsonophagocytosis tests) or protection after *in vivo* challenge (Segura, 2015; Fu et al., 2016; Brockmeier et al., 2018; Feng et al., 2018). Which contains virulence-associated factors such as EF, MRP, SLY and enolase (Wisselink et al., 2001; Esgleas et al., 2009; Segura, 2015) etc. However, the protective efficacy of high levels of antibodies against these proteins remains controversial (Segura, 2015). In fact, a discrepancy between the antibody response and protection has been reported for some other surface antigens of gram-positive bacteria, such as a fibronectin binding protein (Sfb1) of *Streptococcus pyogenes* (McArthur et al., 2004), pneumococcal surface protein A (PspA) (Miyaji et al., 2002) and streptococcal lipoprotein rotamase A (SlrA) of *Streptococcus pneumoniae* (Hermans et al., 2006). This indicating that protective antigens and virulence factors are not necessarily synonymous. However, based on the fact that cell wall-anchored proteins show good target accessibility, we believe that Cba still has potential as a protective antigen for subunit vaccines. Therefore, we evaluated the potential of rCba as a vaccine candidate with the challenge protection experiments. The results showed that rCba was able to induce a strong immune response, but immunization with rCba protein instead of producing protection against mice but enhancing the lethality of mice challenged with SS9-P10. The bacterial load of mouse tissues and blood as well as the histopathological evaluation results also further confirmed that immunization with rCba aggravates the infection of SS in mice. The pathological condition of SS-infected mice deteriorates after immunization with rCba may be the major cause of mortality in mice. Similarly, rCba antigen-specific serum is not protective but increased the mortality rate in mice. These phenomena are similar to those of other bacteria and is known as antibody-dependent enhancement (ADE) (Griffin, 1980; Michl et al., 1983; Astry and Jakab, 1984; Halstead et al., 2010). In addition, it should be noted that we found that even when we increased the challenge concentration of SS9-P10 from approximately 1 × 10^6^ CFU/mouse to approximately 1 × 10^7^ CFU/mouse, the control mice in the active and passive immunization experiments were not all killed. This is inconsistent with the results of previous pathogenicity analysis of SS9-P10 in mice. We speculate that this is probably mainly due to the resistance of the heavier mice being more resistant to the disease (mice were 2–3 weeks old and weighed 21–22 g at the time of first immunization and 8–9 weeks old and weighed 31–33 g when challenged with SS9-P10 after the last immunization).

Specific antibodies prevent extracellular pathogenic microorganisms from harming the health of host. However, owing to co-evolution with their hosts, pathogenic microorganisms have evolved mechanisms to counteract the specific immunity of their hosts, such as hiding relevant binding sites and degrading antibodies (Scherler et al., 2018). Some pathogenic microorganisms can also enhance their ability to invade organisms through antibodies produced by specific mechanisms (Wang et al., 2019) in a phenomenon known as ADE. Currently, there are only a few reports of antibody-enhanced bacterial infections and diseases that support this phenomenon by a mechanism different from viral ADE (Torres et al., 2021). Mechanisms of bacterial ADE include: 1) antibody-mediated serum resistance, 2) virulence enhancement by antibody proteolysis, 3) Antibody-enhanced adhesion, and mediated protection from phagocytosis. However, the potential mechanism of immune rCba-induced ADE production needs to be further investigated.

In conclusion, the surface-anchored protein Cba of SS is a novel virulence factor which is prevalent in pathogenic SS. Although inoculation with rCba was able to produce a strong immune response, the rCba-specific immune response did not mediated protective effects, but increased the pathogenicity of SS in mice. This phenomenon is similar to ADE in bacteria. Therefore, the potential of Cba as a vaccine candidate remains to be further determined.

## Data availability statement

The datasets presented in this study can be found in online repositories. The names of the repository/repositories and accession number(s) can be found below: https://www.ncbi.nlm.nih.gov/, CP015557.1.

## Ethics statement

The animal study was reviewed and approved by Ethics Committee of Animal Subjects of Hunan Agricultural University (Changsha, China).

## Author contributions

XY designed the experiment. PY, QH, KC, and JS conducted animal and laboratory experiments and acquired the data. LY and XY analyzed the data and interpreted the results. LY and PY wrote the manuscript. LY and XY revised the manuscript. YH and DZ prepared the materials and revised the manuscript. All authors contributed to the article and approved the submitted version. 

## References

[B1] Araujo Alves L. Ganguly T. Mattos-Graner R. O. Kajfasz J. Harth-Chu E. N. Lemos J. A.. (2018). CovR and VicRKX regulate transcription of the collagen binding protein cnm of streptococcus mutans. J. Bacteriol. 200 (23), e00141–18. doi: 10.1128/JB.00141-18 30201780 PMC6222205

[B2] Astry C. L. Jakab G. J. (1984). Influenza virus-induced immune complexes suppress alveolar macrophage phagocytosis. J. Virol. 50 (2), 287–292. doi: 10.1128/JVI.50.2.287-292.1984 6708169 PMC255619

[B3] Bonifait L. de la Cruz Dominguez-Punaro M. Vaillancourt K. Bart C. Slater J. Frenette M.. (2010). The cell envelope subtilisin-like proteinase is a virulence determinant for *Streptococcus suis* . BMC Microbiol. 10, 42. doi: 10.1186/1471-2180-10-42 20146817 PMC2832634

[B4] Brockmeier S. L. Loving C. L. Nicholson T. L. Wang J. Peters S. E. Weinert L.. (2018). Use of proteins identified through a functional genomic screen to develop a protein subunit vaccine that provides significant protection against virulent streptococcus suis in pigs. Infection Immun. 86 (3), e00559–e00517. doi: 10.1128/IAI.00559-17 PMC5820948 29203546

[B5] Cabezas A. Costas M. J. Canales J. Pinto R. M. Rodrigues J. R. Ribeiro J. M.. (2022). Enzyme characterization of pro-virulent SntA, a cell wall-anchored protein of *Streptococcus suis*, with phosphodiesterase activity on cyclic-di-AMP at a level suited to limit the innate immune system. Front. Microbiol. 13. doi: 10.3389/fmicb.843068 PMC8981391 35391727

[B6] Esgleas M. Dominguez-Punaro M. L. Li Y. Harel J. Dubreuil J. D. Gottschalk M. (2009). Immunization with SsEno fails to protect mice against challenge with *Streptococcus suis* serotype 2. FEMS Microbiol. Lett. 294 (1), 82–88. doi: 10.1111/j.1574-6968.2009.01551.x 19493012

[B7] Feng L. Niu X. Mei W. Li W. Liu Y. Willias S. P.. (2018). Immunogenicity and protective capacity of EF-tu and FtsZ of *Streptococcus suis* serotype 2 against lethal infection. Vaccine 36 (19), 2581–2588. doi: 10.1016/j.vaccine.2018.03.079 29627237

[B8] Ferrando M. L. Fuentes S. de Greeff A. Smith H. Wells J. M. (2010). ApuA, a multifunctional alpha-glucan-degrading enzyme of streptococcus suis, mediates adhesion to porcine epithelium and mucus. Microbiol. (Reading England) 156 (9), 2818–2828. doi: 10.1099/mic.0.037960-0 20522493

[B9] Fittipaldi N. Segura M. Grenier D. Gottschalk M. (2012). Virulence factors involved in the pathogenesis of the infection caused by the swine pathogen and zoonotic agent *Streptococcus suis* . Future Microbiol. 7 (2), 259–279. doi: 10.2217/fmb.11.149 22324994

[B10] Fu L. Zhao J. Lin L. Zhang Q. Xu Z. Han L.. (2016). Characterization of IgA1 protease as a surface protective antigen of *Streptococcus suis* serotype 2. Microbes infection 18 (4), 285–289. doi: 10.1016/j.micinf.2015.12.005 26774332

[B11] Gottschalk M. Xu J. Calzas C. Segura M. (2010). *Streptococcus suis*: a new emerging or an old neglected zoonotic pathogen? Future Microbiol. 5 (3), 371–391. doi: 10.2217/fmb.10.2 20210549

[B12] Griffin F. M. Jr. (1980). Effects of soluble immune complexes on fc receptor- and C3b receptor-mediated phagocytosis by macrophages. J. Exp. Med. 152 (4), 905–919. doi: 10.1084/jem.152.4.905 7420024 PMC2185974

[B13] Halstead S. B. Mahalingam S. Marovich M. A. Ubol S. Mosser D. M. (2010). Intrinsic antibody-dependent enhancement of microbial infection in macrophages: disease regulation by immune complexes. Lancet Infect. Dis. 10 (10), 712–722. doi: 10.1016/S1473-3099(10)70166-3 20883967 PMC3057165

[B14] Hermans P. W. Adrian P. V. Albert C. Estevão S. Hoogenboezem T. Luijendijk I. H.. (2006). The streptococcal lipoprotein rotamase a (SlrA) is a functional peptidyl-prolyl isomerase involved in pneumococcal colonization. J. Biol. Chem. 281 (2), 968–976. doi: 10.1074/jbc.M510014200 16260779

[B15] Hu Y. Hu Q. Wei R. Li R. Zhao D. Ge M.. (2018). The XRE family transcriptional regulator SrtR in *Streptococcus suis* is involved in oxidant tolerance and virulence. Front. Cell Infect. Microbiol. 8. doi: 10.3389/fcimb.2018.00452 PMC6335249 30687648

[B16] Jelsbak L. Mortensen M. I. B. Kilstrup M. Olsen J. E. (2016). The *In vitro* redundant enzymes PurN and PurT are both essential for systemic infection of mice in *Salmonella* enterica serovar typhimurium. Infection Immun. 84 (7), 2076–2085. doi: 10.1128/IAI.00182-16 PMC4936368 27113361

[B17] Li Q. Liu H. Du D. Yu Y. Ma C. Jiao F.. (2015). Identification of novel laminin- and fibronectin-binding proteins by far-Western blot: Capturing the adhesins of streptococcus suis type 2. Front. Cell. infection Microbiol. 5. doi: 10.3389/fcimb.2015.00082 PMC4644805 26636044

[B18] Li Y. Gottschalk M. Esgleas M. Lacouture S. Dubreuil J. D. Willson P.. (2007). Immunization with recombinant sao protein confers protection against *Streptococcus suis* infection. Clin. Vaccine Immunol. 14 (8), 937–943. doi: 10.1128/CVI.00046-07 17567767 PMC2044494

[B19] Li Y. Martinez G. Gottschalk M. Lacouture S. Willson P. Dubreuil J. D.. (2006). Identification of a surface protein of *Streptococcus suis* and evaluation of its immunogenic and protective capacity in pigs. Infection Immun. 74 (1), 305–312. doi: 10.1128/IAI.74.1.305-312 PMC1346615 16368985

[B20] Lun Z. R. Wang Q. P. Chen X. G. Li A. X. Zhu X. Q. (2007). *Streptococcus suis*: an emerging zoonotic pathogen. Lancet Infect. Dis. 7 (3), 201–209. doi: 10.1016/S1473-3099(07)70001-4 17317601

[B21] McArthur J. Medina E. Mueller A. Chin J. Currie B. J. Sriprakash K. S.. (2004). Intranasal vaccination with streptococcal fibronectin binding protein Sfb1 fails to prevent growth and dissemination of streptococcus pyogenes in a murine skin infection model. Infection Immun. 72 (12), 7342–7345. doi: 10.1128/IAI.72.12.7342-7345 PMC529117 15557665

[B22] Meng X. Shi Y. Ji W. Meng X. Zhang J. Wang H.. (2011). Application of a bacteriophage lysin to disrupt biofilms formed by the animal pathogen *Streptococcus suis* . Appl. Environ. Microbiol. 77 (23), 8272–8279. doi: 10.1128/AEM.05151-11 21984241 PMC3233067

[B23] Meurer M. Öhlmann S. Bonilla M. C. Valentin-Weigand P. Beineke A. Hennig-Pauka I.. (2020). Role of bacterial and host DNases on host-pathogen interaction during *Streptococcus suis* meningitis. Int. J. Mol. Sci. 21 (15), 5289. doi: 10.3390/ijms21155289 32722502 PMC7432635

[B24] Michl J. Unkeless J. C. Pieczonka M. M. Silverstein S. C. (1983). Modulation of fc receptors of mononuclear phagocytes by immobilized antigen-antibody complexes. quantitative analysis of the relationship between ligand number and fc receptor response. J. Exp. Med. 157 (6), 1746–1757. doi: 10.1084/jem.157.6.1746 6854207 PMC2187054

[B25] Miyaji E. N. Ferreira D. M. Lopes A. P. Brandileone M. C. Dias W. O. Leite L. C. (2002). Analysis of serum cross-reactivity and cross-protection elicited by immunization with DNA vaccines against streptococcus pneumoniae expressing PspA fragments from different clades. Infection Immun. 70 (9), 5086–5090. doi: 10.1128/IAI.70.9.5086-5090 PMC128265 12183557

[B26] Nomura R. Nakano K. Naka S. Nemoto H. Masuda K. Lapirattanakul J.. (2012). Identification and characterization of a collagen-binding protein, cbm, in *Streptococcus mutans* . Mol. Oral. Microbiol. 27 (4), 308–323. doi: 10.1111/j.2041-1014.2012.00649 22759315

[B27] Nutravong T. Angkititrakul S. Jiwakanon N. Wongchanthong W. Dejsirilerts S. Nawa Y. (2014). Identification of major *Streptococcus suis* serotypes 2, 7, 8 and 9 isolated from pigs and humans in upper northeastern Thailand. Southeast Asian J. Trop. Med. Public Health 45 (5), 1173–1181. 25417521

[B28] Okura M. Osaki M. Nomoto R. Arai S. Osawa R. Sekizaki T.. (2016). Current taxonomical situation of. Streptococcus suis. Pathog. (Basel Switzerland) 5 (3), 45. doi: 10.3390/pathogens5030045 PMC5039425 27348006

[B29] Sato Y. Okamoto K. Kagami A. Yamamoto Y. Igarashi T. Kizaki H. (2004). *Streptococcus mutans* strains harboring collagen-binding adhesin. J. Dent. Res. 83 (7), 534–539. doi: 10.1177/154405910408300705 15218042

[B30] Scherler A. Jacquier N. Greub G. (2018). Chlamydiales, anaplasma and bartonella: persistence and immune escape of intracellular bacteria. Microbes Infect. 20 (7-8), 416–423. doi: 10.1016/j.micinf.2017.11.002 29162422

[B31] Segura M. (2015). Streptococcus suis vaccines: candidate antigens and progress. Expert Rev. Vaccines 14 (12), 1587–1608. doi: 10.1586/14760584.2015.1101349 26468755

[B32] Segura M. Fittipaldi N. Calzas C. Gottschalk M. (2017). Critical *Streptococcus suis* virulence factors: Are they all really critical? Trends Microbiol. 25 (7), 585–599. doi: 10.1016/j.tim.2017.02.005 28274524

[B33] Staats J. J. Feder I. Okwumabua O. Chengappa M. M. (1997). *Streptococcus suis*: past and present. Vet. Res. Commun. 21 (6), 381–407. doi: 10.1023/a:1005870317757 9266659

[B34] Takamatsu D. Osaki M. Sekizaki T. (2001). Construction and characterization of *Streptococcus suis*-*Escherichia coli* shuttle cloning vectors. Plasmid 45 (2), 101–113. doi: 10.1006/plas.2000.1510 11322824

[B35] Taniyama D. Sakurai M. Sakai T. Kikuchi T. Takahashi T. (2016). Human case of bacteremia due to *Streptococcus suis* serotype 5 in Japan: The first report and literature review. IDCases 6, 36–38. doi: 10.1016/j.idcr.2016.09.011 27689023 PMC5040640

[B36] Torres V. V. L. Coggon C. F. Wells T. J. (2021). Antibody-dependent enhancement of bacterial disease: Prevalence, mechanisms, and treatment. Infect. Immun. 89 (4), e00054–21. doi: 10.1128/IAI.00054-21 33558319 PMC8090947

[B37] Torres V. V. L. Heinz E. Stubenrauch C. J. Wilksch J. J. Cao H. Yang J.. (2018). An investigation into the Omp85 protein BamK in hypervirulent klebsiella pneumoniae, and its role in outer membrane biogenesis. Mol. Microbiol. 109 (5), 584–599. doi: 10.1111/mmi.13990 29873128

[B38] Wang Y. Wang Y. Liu B. Wang S. Li J. Gong S.. (2019). Pdh modulate virulence through reducing stress tolerance and biofilm formation of *Streptococcus suis* serotype 2. Virulence 10 (1), 588–599. doi: 10.1080/21505594.2019.1631661 31232165 PMC6592368

[B39] Wertheim H. F. Nghia H. D. Taylor W. Schultsz C. (2009). *Streptococcus suis*: an emerging human pathogen. Clin. Infect. Dis. 48 (5), 617–625. doi: 10.1086/596763 19191650

[B40] Wisselink H. J. Vecht U. Stockhofe-Zurwieden N. Smith H. E. (2001). Protection of pigs against challenge with virulent *Streptococcus suis* serotype 2 strains by a muramidase-released protein and extracellular factor vaccine. Vet. Rec. 148 (15), 473–477. doi: 10.1136/vr.148.15.473 11334073

[B41] Yi L. Fan Q. Wang Y. Mao C. Li J. Jin M.. (2021). Evaluation of immune effect of *Streptococcus suis* biofilm-associated protein PDH. Vet. Microbiol. 263, 109270. doi: 10.1016/j.vetmic 34749282

[B42] Zhang A. Chen B. Li R. Mu X. Han L. Zhou H.. (2009). Identification of a surface protective antigen, HP0197 of *Streptococcus suis* serotype 2. Vaccine 27 (38), 5209–5213. doi: 10.1016/j.vaccine.2009.06.074 19596417

[B43] Zhang A. Yang M. Hu P. Wu J. Chen B. Hua Y.. (2011). Comparative genomic analysis of *Streptococcus suis* reveals significant genomic diversity among different serotypes. BMC Genomics 12, 523. doi: 10.1186/1471-2164-12-523 22026465 PMC3227697

[B44] Zhang H. Ma Z. Li Y. Zheng J. Yi L. Fan H.. (2013). Identification of a novel collagen Ukrainian-Binding protein from *Streptococcus suis* serotype 2. Vet. J. 197 (2), 406–414. doi: 10.1016/j.tvjl.2013.01.030 23465548

[B45] Zhang X. Jiang X. Yang L. Fang L. Shen H. Lu X. (2015). DnaJ of *Streptococcus suis* type 2 contributes to cell adhesion and thermotolerance. J. Microbiol. Biotechnol. 25 (6), 771–781. doi: 10.4014/jmb.1408.08085 25537722

[B46] Zheng H. Qiu X. Roy D. Segura M. Du P. Xu J.. (2017). Genotyping and investigating capsular polysaccharide synthesis gene loci of non-serotypeable *Streptococcus suis* isolated from diseased pigs in Canada. Vet. Res. 48 (1), 10. doi: 10.1186/s13567-017-0417-6 28219415 PMC5322794

