# Supplementary material for: Novel virulence factor Cba induces antibody-dependent enhancement (ADE) of Streptococcus suis Serotype 9 infection in a mouse model
Source: Front Cell Infect Microbiol. 2023 Feb 21;13:1027419. doi: 10.3389/fcimb.2023.1027419 (PMC9989217; doi:10.3389/fcimb.2023.1027419)
Supplement: Supplementary file 1 [file DataSheet_1.pdf]

**Additional Table 1**

**Summary of whole genome sequenced SS strains containing the cba gene deposited in GenBank**

| Strains | Serotype | Source of strains | Sensitive animals / pathogenicity | Cite |
|---|---|---|---|---|
| SS GZ0565 | 9 | Sick pigs | Pigs and mice/Weaker | LAI L,et al.2017 |
| SS DN13 | 9 | Sick pigs | Pigs and mice/Weaker | Hu,et al.2019 |
| SS D12 | 9 | Pigs | Unknown | ZHANG,et al.2011 |
| SS DNC15 | 16 | Sick pigs | Unknown | - |
| SS LSS42 | Unknown | Sick pigs | Unknown | - |
| SS SC183 | Unknown | Pigs | Unknown | WANG,et al.2013 |
| SS M106471_S40 | 30 | Pigs | Unknown | - |
| SS TL13 | 16 | Pigs | Unknown | WANG,et al.2013 |
| SS DAT741 | Unknown | Bovine | Unknown | - |
| SS DAT300 | ST115 | Pigs | Pigs and human/Weaker | Minowa-Nozawa A,et al.2020 |
| SS INT-01 | 3 | Sick pigs | Pigs/Stronger | Park SY, et al.2021 |
| SS 2018WUSS151 | Unknown | Sick pigs | Unknown | - |
| SS TJS75 | 2 | Pigs | Unknown | - |
| SS ISU2514 | 2 | Sick pigs | Pigs/Stronger | Nicholson TL,et al.2021 |
| SS ST3 | 3 | Sick pigs | Unknown | Hu,et al.2011 |
| SS 13-00283-02 | 7 | Sick pigs | Pigs/Stronger | Bunk B,et al.2021 |
| SS STC104 | 2 | Patient | Pigs and human/Unknown | - |
| SS STC90 | 2 | Patient | Pigs and human/Unknown | - |
| SS STC86 | 2 | Patient | Pigs and human/Unknown | - |
| SS STC85 | 2 | Patient | Pigs and human/Unknown | - |
| SS STC84 | 2 | Patient | Pigs and human/Unknown | - |
| SS STC83 | 2 | Patient | Pigs and human/Unknown | - |
| SS STC81 | 2 | Patient | Pigs and human/Unknown | - |
| SS STC80 | 2 | Patient | Pigs and human/Unknown | - |
| SS STC78 | 2 | Patient | Pigs and human/Unknown | - |
| SS MSUI060 | 2 | Sick pigs | Pigs and mice/Stronger | Fittipaldi N,et al.2011 |
| SS YB51 | 3 | Pigs | Unknown | KAICHENG W, et al.2013 |
| SS D9 | 7 | Pigs | Unknown | ZHANG,et al.2011 |

Bunk, B., Jakóbczak, B., Florian, V., Dittmar, D., Mäder, U., Jarek, M., et al. (2021). Complete genome sequences of *Streptococcus suis* pig-pathogenic strains 10, 13-00283-02, and 16085/3b. *Microbiol. resource announcements* 10 (2), e01137–e01120. doi: 10.1128/MRA.01137-20

Fittipaldi, N., Xu, J., Lacouture, S., Tharavichitkul, P., Osaki, M., Sekizaki, T., et al. (2011). Lineage and virulence of *Streptococcus suis* serotype 2 isolates from north *America. Emerging Infect. Dis.* 17 (12), 2239–2244. doi: 10.3201/eid1712.110609

Hu, P., Yang, M., Zhang, A., Wu, J., Chen, B., Hua, Y., et al. (2011). Complete genome sequence of *Streptococcus suis* serotype 3 strain ST3. *J. bacteriol.* 193 (13), 3428–3429. doi: 10.1128/JB.05018-11

Lai, L., Dai, J., Tang, H., Zhang, S., Wu, C., Qiu, W., et al. (2017). *Streptococcus suis* serotype

9 strain GZ0565 contains a type VII secretion system putative substrate EsxA that contributes to bacterial virulence and a vanZ-like gene that confers resistance to teicoplanin and dalbavancin in streptococcus agalactiae. *Vet. Microbiol*. 205, 26–33. doi: 10.1016/j.vetmic.2017.04.030

Minowa-Nozawa, A., Nozawa, T., Takamatsu, D., Yoshida, A., Murase, K., Kikuchi, T., et al. (2020). Complete genome sequences of two *Streptococcus suis* strains isolated from asymptomatic pigs. *Microbiol. resource announcements* 9 (47), e01142-20. doi: 10.1128/MRA.01142-20

Nicholson, T. L., Waack, U., Anderson, T. K., Bayles, D. O., Zaia, S. R., Goertz, I., et al. (2021). Comparative virulence and genomic analysis of *Streptococcus suis* isolates. *Front. Microbiol.* 11. doi: 10.3389/fmicb.2020.620843

Park, S. Y., Kim, I. H., Yu, H. J., Paik, H. R., Son, J. S., and Kim, J. H. (2021). Complete genome sequence of serotype 3 *streptococcus suis* INT-01, isolated from a domestic pig in Korea. *J. Anim. Sci. Technol.* 63 (3), 662–665. doi: 10.5187/jast.2021.e47

Wang, K., Chen, J., Yao, H., and Lu, C. (2013). Whole-genome sequence of *Streptococcus suis* serotype 3 strain YB51. *Genome announcements* 1 (6), e00884–e00813. doi: 10.1128/genomeA.00884-13

Wang, K., Yao, H., Lu, C., and Chen, J. (2013). Complete genome sequence of *Streptococcus suis* serotype 16 strain TL13. *Genome announcements* 1 (3), e00394–e00313. doi: 10.1128/genomeA.00394-13

Zhang, A., Yang, M., Hu, P., Wu, J., Chen, B., Hua, Y., et al. (2011). Comparative genomic analysis of *Streptococcus suis* reveals significant genomic diversity among different serotypes. *BMC Genomics* 12, 523. doi: 10.1186/1471-2164-12-523
